# Variation in flavonoid and antioxidant activities of *Pyrrosia petiolosa* (Christ) Ching from different geographic origins

**DOI:** 10.3389/fpls.2023.1173489

**Published:** 2023-04-14

**Authors:** Jianhua Chen, Shan Ning, Xuan Lu, Wei Xiang, Xiao Zhou, Yuanyuan Bu, Liangbo Li, Rongshao Huang

**Affiliations:** ^1^ College of Pharmacy, Guangxi University of Chinese Medicine, Nanning, China; ^2^ Key Laboratory of Protection and Utilization of Traditional Chinese Medicine and Ethnic Medicine Resources, Education Department of Guangxi, Nanning, China; ^3^ Guangxi Institute of Chinese Medicine & Pharmaceutical Science, Nanning, China; ^4^ College of Horticulture, Hunan Agricultural University, Hunan, China

**Keywords:** *Pyrrosia petiolosa*, flavonoids, antioxidant activity, metabolomics, UPLC-MRM/MS

## Abstract

*Pyrrosia petiolosa* (Christ) Ching has both medicinal and health benefits in China. The potential antioxidant activities of *P. petiolosa*, which are mainly attributed to its flavonoids, have attracted much attention in recent years. The present study aimed to determine the concentration of flavonoid components and evaluate the relative antioxidant activities of *P. petiolosa* from different geographic origins using a UPLC-MRM-MS-based metabolomics approach. In total, 97 flavonoid components were identified, and their concentrations in the samples from different geographic locations showed significant variation. Thirteen flavonoid components were identified as potential biomarkers for distinguishing between the two major regions, Guizhou (GZ) and Guangxi (GX). The GZ group showed higher total flavonoid content, free radical scavenging activities, and ferric reducing antioxidant power. The well positive correlations were found between the antioxidant capacities and some flavonoid markers. The ecogeographic factors, namely altitude and longitude, play a crucial role in the difference of antioxidant activities and flavonoids concentration. These results indicate that *P. petiolosa* is rich in flavonoid compounds and is a promising source of natural antioxidants, providing a basis for the quality control of *P. petiolosa*.

## 1 Introduction

Flavonoids are the most common phenolic compounds found in plants and contribute to improving human health and reducing the risk of chronic diseases. They are known to possess anti-inflammatory, antioxidant, anti-inflammatory, anti-aging, and immunomodulatory properties, and are widely utilised in nutraceutical, medicinal, and health products owing to their plant origin and low toxicity (Shen et al., 2022). Antioxidant activity is the main biological effect of flavonoids, and therefore has been extensively studied. Various mechanisms underlying this antioxidant activity have been uncovered, including inhibition of oxidases, activation of antioxidant enzymes, scavenging of reactive oxygen species, alleviation of oxidative stress, and metal chelation (Williamson et al., 2018). Appropriate intake of flavonoid-rich foods or other functional products with antioxidant effects has beneficial effects on human health by reducing oxidative stress injury from free radicals (Rana et al., 2022). Flavonoids are highly relevant in the discovery and development of natural antioxidants, and as such much research in this area has focussed on flavonoids as potentially useful compounds.

*Pyrrosia petiolosa* (Christ) Ching, a perennial herb fern, belongs to the *Pyrrosia* genus in the family Polypodiaceae and is mainly found across the western region of China. *P. petiolosa* has a long history of medicinal use in China and has proven effective in the clinic for treating pyelonephritis, chronic bronchitis, and urinary calculi (Xiao et al., 2017; Lang et al., 2021). Previous studies have shown that *P. petiolosa* exhibits strong antioxidant activity, which is mainly attributed to the high concentration of flavonoid components (Cheng et al., 2014). Bioactive flavonoid compounds derived from *P. petiolosa* are a potential source of natural antioxidants that positively influence oxidative stress (Zhou and Wang, 2022). Although some flavonoid components, such as kaempferol, mangiferin, and quercetin, have been identified in *P. petiolosa* (Cui et al., 2016), more detailed metabolic profiles of flavonoids in *P. petiolosa* are lacking. Furthermore, there is still little direct evidence regarding the variation in the concentration of these flavonoids, and little is known about whether the overall antioxidant activity is correlated with the levels of individual flavonoids.

The biological activity and clinical efficacy of herbs or functional foods is highly dependent on their secondary metabolic composition, and the concentration of active compounds is often used as an important indicator of quality. Plant secondary metabolites are the result of the plant’s continued interaction with the environment over the course of evolution (Moore et al., 2014). Compared to the primary metabolism, secondary metabolic reactions are therefore more susceptible to the external environment. Numerous recent studies have shown that the chemical composition of various plants is significantly influenced by their geographical location (Li et al., 2020b; Liu et al., 2020). Environmental and edaphic factors, such as latitude and longitude, altitude, climate, and various soil physicochemical properties, also indirectly affect the secondary metabolites of plants (Guo et al., 2013). Such local specificity in the secondary metabolite profiles of plants is advantageous since it confers protection and adaptive advantages against environmental stress (Mahajan et al., 2020). There is little information regarding the variation in chemical components, especially flavonoids, in *P. petiolosa* from different geographical locations and environmental factors, resulting in uneven quality of *P. petiolosa* when incorporated.

To explore differential metabolites in *P. petiolosa* with different geographical origins, appropriate chemical analysis methods are particularly important. Recently, targeted metabolomics has successfully detected the differences between the levels of small-molecule metabolites in biological systems by simultaneous qualitative or quantitative analysis. Therefore, targeted metabolomics is increasingly recognized as an effective tool for assessing the quality of herbs or functional foods and determining their geographical origin (Mais et al., 2018). Liquid chromatography-tandem mass spectrometry (LC-MS/MS) has frequently been used in metabolomics studies because of its high sensitivity and specificity. Many biomarkers identified through metabolomics technologies are not only used to systematically study the mechanisms of functional components but also to explore the differences between various plant categories or similar species, diverse geographic origins, or different growth and cultivation conditions of herbs or functional foods (Zhang et al., 2020).

The objectives of the present study were to (i) compare the flavonoid-related profiles between *P. petiolosa* from different geographical origins and screen for flavonoid-related biomarkers by quantifying the flavonoid compositions using targeted metabolomics techniques, (ii) evaluate the antioxidant activity of *P. petiolosa* from different geographical origins and correlate the antioxidant activity with individual biomarkers, and (iii) determine the correlation between environmental factors, antioxidant activity, and major flavonoid markers.

## 2 Materials and methods

### 2.1 Plant materials

**Figure 1:**
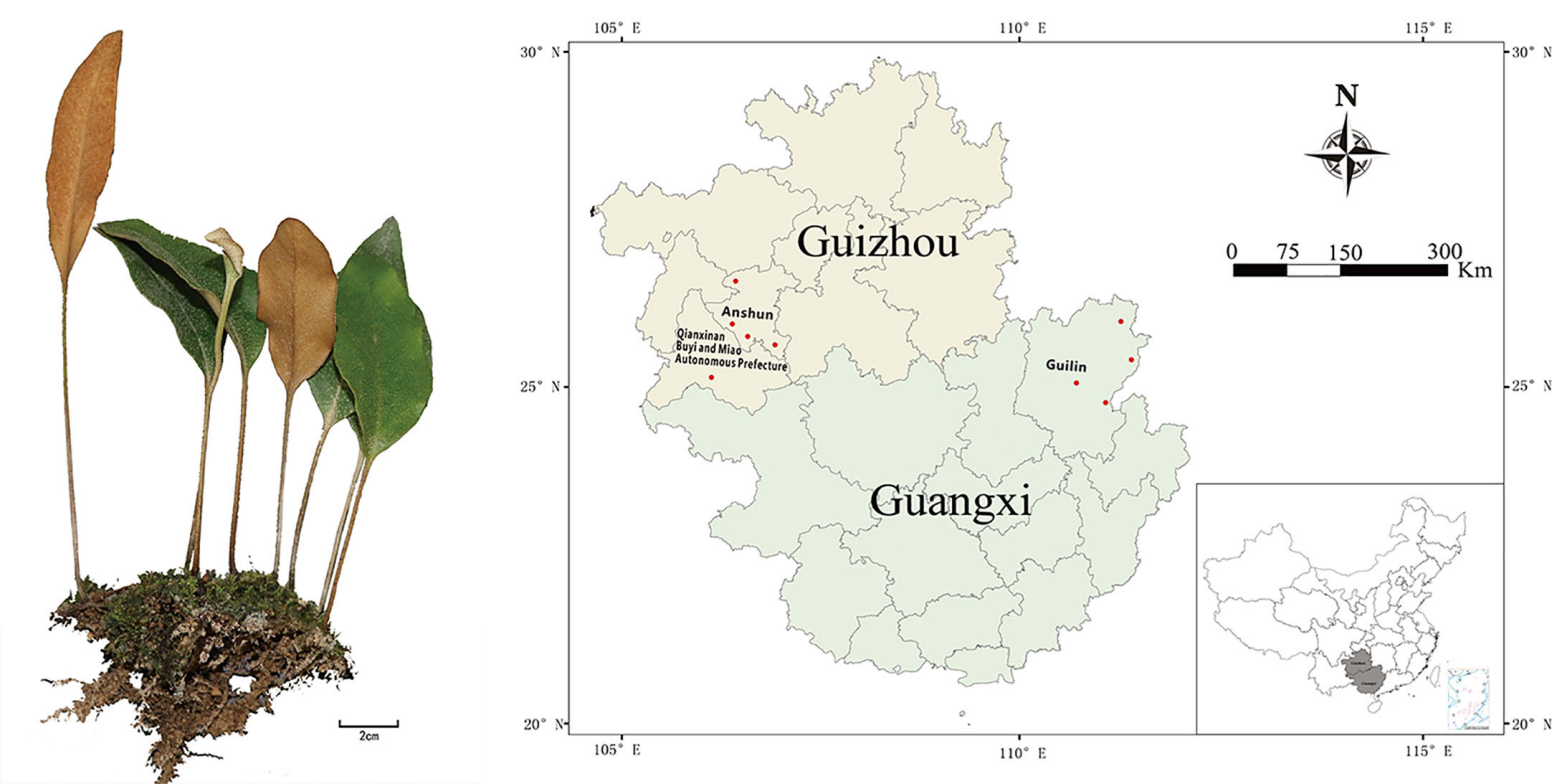
Geographical locations of *P. petiolosa* samples. For names of the numbered positions and ecogeographic information see **Supplementary Table 1**.

A total of 27 batches of *P. petiolosa* samples were collected from natural habitats in western China, which represents the primary geographical region from which the medicinal products of *P. petiolosa* in China are derived. Samples were taken from nine locations distributed across two major regions, Guizhou (GZ) and Guangxi (GX) (**Figure 1**). Representative plant samples were collected in triplicate at each location. All samples were authenticated by the School of Pharmacy of the Guangxi University of Chinese Medicine. The altitude, longitude, and latitude of each sampling location were recorded, and original datasets recording the annual climatological values (including precipitation, average temperature, sunshine, and relative humidity) of each geographical location were obtained from the National Meteorological Information Center (**Supplementary Table 1**).

### 2.2 Sample preparation

All samples were ground into a powder using a grinding mill and sieved through a 50−mesh sieve. The powders from each replicate were thoroughly mixed for qualitative analysis. For ultra-performance liquid chromatography-tandem mass spectrometry (UPLC-MS/MS) analysis, 25 mg of the powder was extracted by ultrasonication in 500 μL of 70% methanol solution. After centrifugation at 12,000 × g for 5 min, the supernatants were collected and filtered through a 0.22 μm membrane, then transferred to sample vials. For antioxidant assays and total flavonoid content (TFC) determination, powder of plant leaves (2 g) was extracted with 100 mL of 70% methanol by heat reflux extraction for 1 h, and ultrasonic extraction was then carried out at 30°C for 30 min. The power output of the sonicator was set at 100 kHz. After filtration, the methanol extracts were concentrated under vacuum and lyophilised. Methanol extracts of each sample were prepared at and stored at 20°C until further use.

### 2.3 UPLC-MS analysis

UPLC separation was performed in an ExionLC™ AD system equipped with an ACQUITY UPLC HSS T3 C18 (1.8 µm, 100 mm×2.1 mm i.d., Waters, Milford, Massachusetts, USA) column maintained at 40 °C. The injection volume for each sample was 2 µL. Elution was carried out using a mobile phase of 0.05% formic acid in water (eluent A) and acetonitrile (eluent B). The flow rate was set to 0.35 ml/min. An elution gradient program was performed as follows: 0–1 min, 10–20% B; 1–9 min, 20–70% B; 9–12.5 min, 70–95% B; 12.5–13.5 min, 95% B; 13.6–15 min, 95–10% B.

MS analysis was performed using an AB SCIEX QTRAP 5500 mass spectrometer equipped with an ESI source under both negative (−) and positive (+) ion modes. The operating parameters were as follows: ion source, turbo spray; source temperature, 550°C; ion spray voltage (IS), 5.5 kV for positive ion mode and -4.5 kV for negative ion mode; curtain gas, 35 psi.

For qualitative analysis, flavonoid compounds were annotated using primary and secondary MS data based on the self-built metware database (MWDB). For quantitative analysis, the concentrations of flavonoid compounds in *P. petiolosa* were quantified using the standard addition method. Stock solutions were prepared by dissolving the standards in methanol. The working solutions of the individual flavonoid standards in the concentration range 0.5 nmol/L—1000 nmol/L were prepared by serial half dilutions. The multiple-reaction monitoring (MRM) mode in QQQ MS was used, and the MRM transitions for each metabolite were determined. The collision energy (CE) and declustering potential (DP) were optimised for the individual MRM transitions to produce maximal signals. Optimised UPLC-MS/MS was applied to collect MS/MS spectra of individual analyte standards in a heartwood sample. Optimised UPLC-MS was used to collect mass spectral peak intensity data for the quantitative signals corresponding to the concentrations of the individual standards. The standard curves for the different compounds were plotted using the external standard concentration as the horizontal coordinate and the external standard peak area as the vertical coordinate. The linear equations and correlation coefficients of the standard curves for the substances tested in this study are listed in **Supplementary Table 2**. The mass spectral peaks detected for each substance in the different samples were corrected for quantitative accuracy based on information about the retention time and peak shape of the standards. All samples were quantified, and the peak area represented the relative content of the corresponding substance, which was substituted into the linear equation to obtain the final quantitative results for all flavonoid compounds in all samples.

### 2.4 Determination of antioxidant activity

#### 2.4.1 2,2-diphenyl-1-picryhydrazyl radical scavenging capacity

DPPH radical scavenging capacity was measured as described by Olszowy and Dawidowicz (2016), with some modifications. DPPH radical was dissolved in methanol. In a 96-well microplate, 100 µL DPPH radical solution was mixed with 100 µL methanol extract of each sample (0.1 mg/mL) or methanol as a negative control. The mixtures were incubated in the dark for 30 min and the absorbance was read at 515 nm. The results are expressed as mg of Trolox equivalents (TE) per gram of dry weight (mg TE/g DW), with a calibration curve of Trolox within the concentration range of 5–25 μg/mL (calibration curve: y = 3.1324 x- 0.699, *R^2^* = 0.997).

#### 2.4.2 2,2’-azino-bis(3-ethylbenzothiazoline-6-sulfonic acid) radical cation scavenging activity

ABTS scavenging activity was assayed as described by Vifta and Luhurningtyas (2019). ABTS solution was prepared by mixing 7 mM ABTS solution with 2.45 mM ammonium persulphate (final concentration) and kept in the dark for at least 16 h at room temperature. The working solution was prepared by diluting ABTS solution with phosphate buffer (pH 7.4) to an absorbance of 0.750 at 734 nm. In 96-well microplates, 10 µL of sample extracts (0.5 mg/mL) were mixed with 190 µL ABTS working solution and allowed to react at 30°C for 20 min. The absorbance was measured at 734 nm. The mixtures containing ABTS working solution and methanol were used as blanks. The results are expressed as mg of TE per gram of dry weight (mg TE/1 g DW), with a calibration curve of Trolox within the concentration range of 5–25 μg/mL (calibration curve: y = 0.625 x + 0.4697, *R^2^* = 0.9999).

#### 2.4.3 Ferric reducing antioxidant power assay

The FRAP assay was performed as described by Lin et al. (2022), with some modifications. The working FRAP solution was freshly prepared by mixing acetate buffer (300 mM, pH3.6), 10 mM 2, 4, 6-Tris (2-pyridyl)-s-triazine (TPTZ) solution in 40 mM HCl, and 20 mM ferric chloride solution at a ratio of 10:1:1, and was then incubated at 37°C before use. In 96-well microplates, 5 µL sample extracts (2.0 mg/mL) were mixed with 190 µL FRAP working solution and left in the dark for 30 min at room temperature. Mixtures containing the working solution and distilled water were used as blanks. Absorbance was measured at 590 nm. The results are expressed as nmol of TE per gram of dry weight (mg TE/1 g DW). with a calibration curve of Trolox within the concentration range of 0.4 – 3.6 μg/mL (calibration curve: y = 0.3651 x- 0.0514, R2 = 0.9909).

### 2.5 Determination of total flavonoid content

TFC was determined using the aluminium chloride colorimetric method as described by Zhang et al. (2022), with some modifications. In 96-well microplates, 50 µL of methanol extract (0.5 mg/mL) and 15 µL of 5% (w/v) NaNO_2_ were added. After 6 min, 30 µL of 10% (w/v) AlCl_3_ was added to the mixture, followed by 105 µL of 1 mol/L NaOH, and absorbance was measured at 510 nm. The TFC was calculated from a calibration curve of rutin within the concentration range of 0.2 – 1 mg/mL (calibration curve: y = 1.0843 x - 0.0158, *R^2^* = 0.9989). The results are expressed as milligrams of rutin equivalents (RE) per 100 g dry weight of the sample (mg RE/100 g DW).

### 2.6 Data analysis

The final experimental data are presented as the mean ± standard deviation of three independent experiments. One-way analysis of variance (ANOVA) was applied using IBM SPSS Statistics version 22 (SPSS, Chicago) to assess the differences between mean values, followed by Duncan’s multiple comparisons with a 95% confidence level. OriginLab Origin Pro software version 2021b (OriginLab, Northampton, MA, U.S.) was used for graph production.

Hierarchical cluster analysis (HCA) was performed to investigate similarities and relationships among different habitats. The concentrations of each flavonoid compound were imported into SIMCA software (14.1 version, Umetrics AB, Umea, Sweden) to perform principal component analysis (PCA) and orthogonal projections to latent structures discriminant analysis (OPLS-DA). Variable Importance in Projection (VIP) scores were calculated using the OPLS-DA model. An unpaired *t*-test was used to perform univariate analyses of the flavonoid compounds. Flavonoid compounds with VIP ≥ 1.5, fold change (FC) ≥ 1, or FC ≤ 0.5, and p < 0.05, were identified as potential differential compounds. Pearson’s correlation coefficient was calculated between differential flavonoid compounds and antioxidant capacity, and a corresponding heatmap was drawn using the R software (www.r-project.org/). Redundancy analysis was carried out to assess the relationship between the environment and differential flavonoid compounds in different samples using Canoco software (5.0 version, Petr Šmilauer).

## 3 Results and discussion

### 3.1 Flavonoid profiles and their concentration in *Pyrrosia petiolosa*

**Figure 2:**
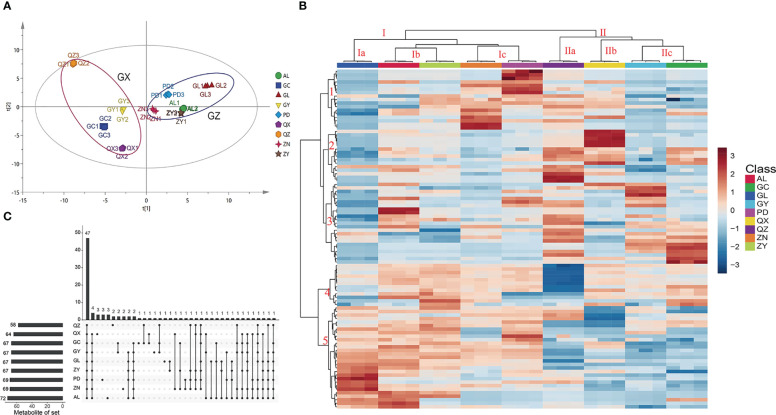
Multivariate statistical analysis of *P. petiolosa* from different geographic origins based on UPLC-MS profiles. **(A)** Score plot of principal component analysis. **(B)** Heatmap of 97 flavonoid components in *P. petiolosa* samples from different geographic origins; the heatmap colours indicate the concentration of each metabolite, from low (blue) to high (red). **(C)** The UpSet diagram shows the overlapping and origin-specific flavonoid components in *P. petiolosa* samples.

The flavonoid characteristics are one of the most important traits affecting the medicinal value of *P. petiolosa* (Yang et al., 2003). In present study, a total of 97 flavonoids were identified, and the flavonoid metabolic profile can be represented as chromatographic peaks in the total ion chromatogram (TIC) (**Supplementary Figure 1**). These flavonoids were quantified using calibration standards for each samples, and their concentration levels of each flavonoid across the samples are listed in **Supplementary Materials 2**. Differences in the flavonoid content of *P. petiolosa* from different geographical origins were discriminated using an unsupervised PCA model (**Figure 2A**). In the PCA score plot, the first and second principal components (PC1 and PC2) explained 30.9% and 16.2% of the total variance, respectively. Nine sampling locations formed two distinct groups, respectively, which corresponded to the two regions (GX and GZ) from which the *P. petiolosa* samples were obtained, suggesting significant differences between the flavonoid profiles of the two regions.

The differences in flavonoid concentration among samples from different geographical origins and the similarity among the biological replicates, can be seen in the heatmap representing flavonoid content (**Figure 2B**). Furthermore, HCA (hierarchical clustering analysis) according to the Euclidean distance suggested that all samples can be divided to two clusters (I and II) on the abscissa axis, in agreement with the results of PCA. The cluster I is again sub divided into three sub clusters where the first subcluster (Ia) includes GL; second sub cluster (Ib) consists of AL and ZN; third sub cluster (Ic) consists of ZN and PD. The second main cluster (II) is also sub divided into three sub clusters. The first sub cluster (IIa) includes QZ; second sub cluster (IIb) includes QX. The third sub cluster (IIc) consists of GY and GC. Similarly, according to HCA, the samples from different locations in the same regions were also clustered separately. Five major categories were identified on the ordinate axis based the accumulation of flavonoids. The flavonoids in category 1 accumulated at the highest levels in ZN and PD, and at the lowest levels GL. In category 2, flavonoids accumulated at the highest levels in QZ and QX, and the lowest levels in GL and GY. Flavonoids in category 3 accumulated at the highest levels in QZ and GL, and the lowest levels in GL. Flavonoids in category 4 accumulated at the highest levels in ZY, followed by AL and ZN, and the lowest levels in QZ. Flavonoids in category 5 showed significant accumulation in the samples of GZ region (GL, ZN, ZY, AL, PD), whereas less accumulation in GX region (GC, GY, QX, QZ). Thus, together PCA and HCA analyses suggested that the samples of GZ region and GX region had obviously distinct flavonoids profiles.

The UpSet diagram shows overlapping and origin-specific flavonoids in the different samples (**Figure 2C**). The amount of flavonoids detected was highest in the AL and lowest in the QZ. It is clear that there is a rich diversity among *P. petiolosa* samples of different geographical origins, both in terms of flavonoid composition and flavonoid concentration. Of these components, some flavonoids have been detected in samples from only a few origins (**Figure 2C**). Meanwhile, compared with the variation of concentration in *P. petiolosa* among origins, some components of the flavonoid (such as astragalin, afzelin, kaempferol, vitexin and so on) were approximately several times as variable (**Supplementary Materials 2**). The genetic changes and environmental factors are the main causes of this phenotypic diverse (Santos et al., 2019; Li et al., 2020a). Necessitates further study is required to explore how the flavonoid diversity of *P. petiolosa* is affected by the main environmental factors, genetic factors, and interactions between them. There were 47 flavonoids present in all samples (**Figure 2C**). Of these, eight flavonoids, (-)-catechin, astragalin, baimaside, hyperoside, orientin, afzelin, rutin, and scutellarin, were present at particularly high levels in all samples and are considered to be the main flavonoid constituents of *P. petiolosa*. Most of these main flavonoids are flavone glycosides. Hence, our findings are in close agreement with previous reports that the flavonoid species in *P. petiolosa* are mainly flavonoids, flavonols, dihydroflavonoids, and flavone glycosides (Wang et al., 2006; Huh and Sung, 2012). However, the absolute quantification of such a large number of flavonoid constituents has not previously been reported and has been presented for the first time in the present study. Quantification is important since it provides a theoretical basis for the quality control of *P. petiolosa* from different sources.

### 3.2 Metabolomics analysis of *Pyrrosia petiolosa* samples from different geographic origins

**Figure 3:**
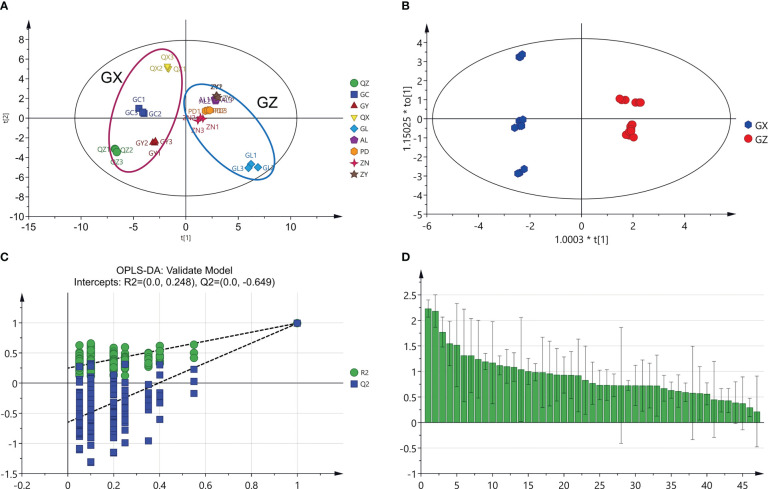
Multivariate statistical analysis of two groups of *P. petiolosa* based on the concentration of 47 common flavonoids. **(A)** Principal component analysis (PCA) score plot; **(B)** Orthogonal partial least square discriminant analysis (OPLS-DA) score plot; **(C)** results of permutation testing of the OPLS-DA model with 200 repetitions; **(D)** VIP score plot of the candidate flavonoids markers for *P. petiolosa* samples.

Metabolomics analysis provides a new perspective to understand the metabolic variations in traditional herbs grown in different geographical environments, and likewise contributes to the differentiation of high-quality herbs (Tang et al., 2021). In the present study, metabolomic analysis was used to discriminate between *P. petiolosa* samples from different geographical origins based on the content of 47 common flavonoid constituents. The PCA score plot showed that all samples were clearly separated into two distinct groups, consistent with the two large distribution regions of *P. petiolosa*, even when only the 47 common flavonoid constituents were assessed (**Figure 3A**). OPLS-DA was then performed to further screen for the potential markers responsible for separating samples into the two groups (**Figure 3B**). OPLS-DA, a supervised model, is a classical method used in metabolomics research that can reduce system noise and extract variable information, thereby enabling more accurate discovery of inter-group differential metabolites (Guo et al., 2018). The OPLS-DA results revealed that the flavonoid composition samples from GZ and GX were significantly different (**Figure 3C**). Model parameters [R^2^Y(cum) = 0.996 and Q^2^ (cum) = 0.992)] indicate that the model was stable and performed well in terms of fitness and prediction (Li et al., 2021). Subsequently, a permutation test (n=200) was performed to verify the model using the previous methods. As shown in **Figure 2C**, R^2^ and Q^2^ were greater than the original value, and the Q^2^ intercept value was lower than 0 [R^2^ = (0.0, 0.248), Q^2^ = (0.0, –0.649)], indicating that the model verification was successful because the original model was not over-fitted. The OPLS-DA model generates VIP scores and can be used to screen for differential metabolites. A set of 15 flavonoid constituents were found to have a VIP score >1.5 (**Figure 3D**).

### 3.3 Screening chemical markers

**Figure 4:**
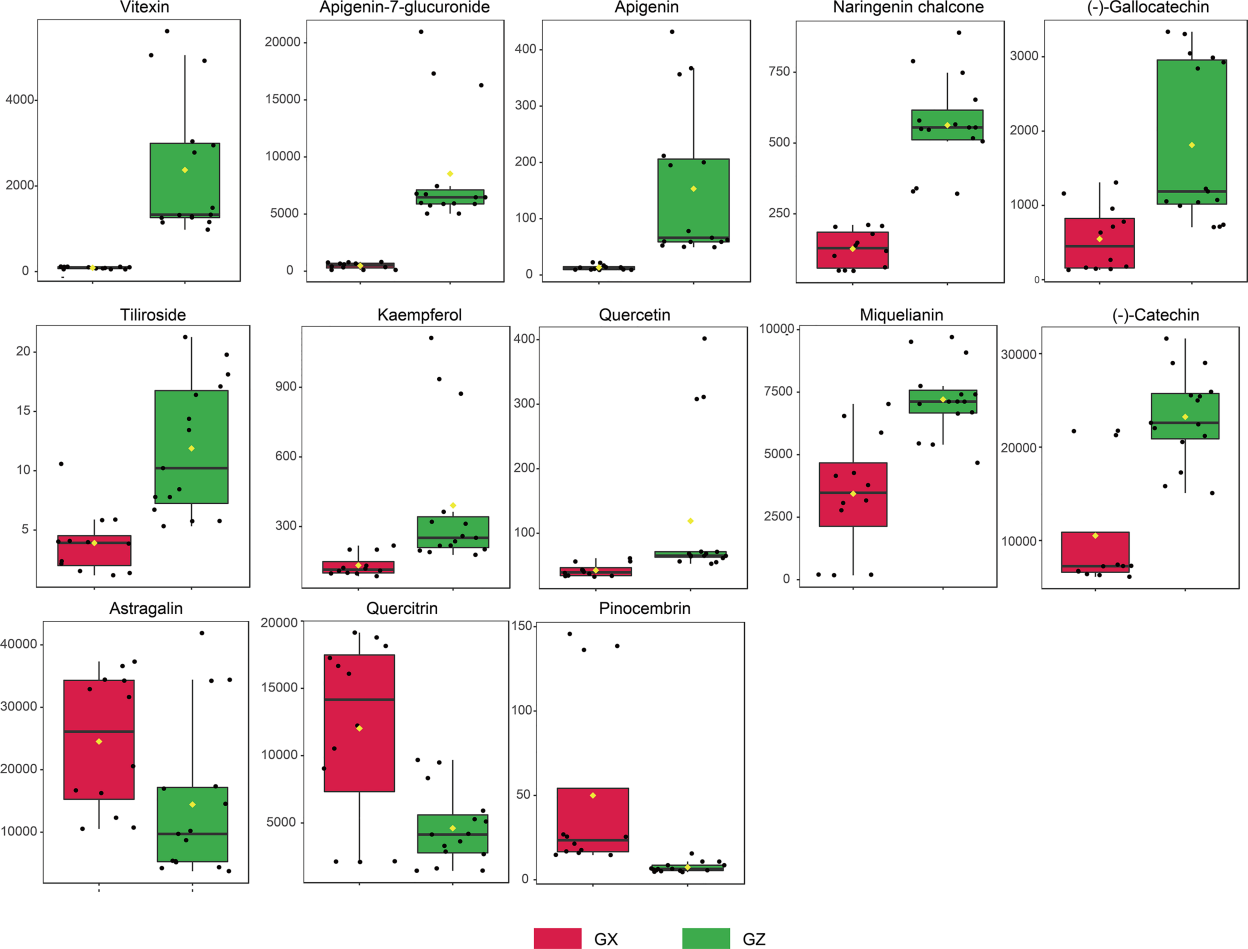
Concentration variation of flavonoid markers in *P. petiolosa* from different origins. Y-axis unit is nmol/g.

Potential marker compounds in the two groups were selected using multivariate (OPLS-DA), univariate (ANOVA), and fold change (FC) analysis. A strict screening threshold was set such that metabolites with VIP ≥ 1.5, fold change (FC) ≥ 1, or FC ≤ 0.5, and p < 0.05, were identified as potential marker compounds. Overall, 13 flavonoid constituents showed significant differences between the GZ and GX groups (**Supplementary Table 3**). Boxplots show that the concentration of all flavonoid markers, except for astragalin, quercitrin, and pinocembrin, was significantly upregulated in the GZ group. In the GZ group, the presence of (-)-catechin was the highest, followed by that of miquelianin and apigenin-7-glucuronide (**Figure 4**).

### 3.4 *In vitro* antioxidant activities and total flavonoids analysis

**Figure 5:**
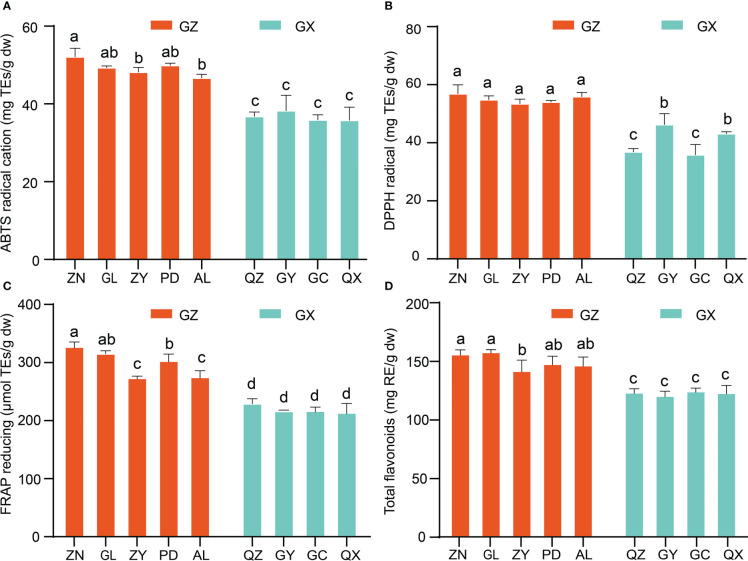
In vitro antioxidant activities and total flavonoid content of *P. petiolosa* samples from different geographical origins. **(A)** ABTS radical scavenging activity. **(B)** DPPH radical scavenging ability. **(C)** Ferrous ions chelating activity. **(D)** Total flavonoid content. Error bars indicate the standard error of the mean values. Different letters above the bars indicate significant differences (p < 0.05).

In the present study, *in vitro* assays of the antioxidant activity of *P. petiolosa* were employed based on different mechanisms, including antioxidant capacity (DPPH and ABTS free radical scavenging activities) and FRAP. Although the antioxidant activity of *P. petiolosa* extract has previously been reported (Hsu, 2008), a comparison of the antioxidant activity of *P. petiolosa* derived from different geographical origins has not yet been conducted. The results obtained are shown in **Figures 5A–D**. Comparing samples from the two main distribution regions, the ABTS value, DPPH value, and FRAP value of the GZ group were all significantly higher than those of GX group, (p > 0.05). Samples from location ZN had the most potent DPPH radical scavenging ability (57.14 ± 3.14 mg TE/g dw), ABTS radical cation scavenging activity (52.32 ± 2.24 mg TE/g dw), and FRAP (328.12 ± 10.75 mg TE/g dw), indicating that ZN is probably the location best suited for the growth of *P. petiolosa* for medicinal use. Similar to the antioxidant activity, a higher TFC was observed in the GZ group compared to that in the GX group. Similar data have been reported by other authors, where some fruits and herbs collected from different geographical origins showed significant differences in both TFC and antioxidant activities (Al-Mekhlafi et al., 2021; Sheng et al., 2021). Overall, the results of the antioxidant activity assessment were consistent with those for the TFC and from the multivariate analysis of the flavonoid constituents of *P. petiolosa* from different geographical areas, suggesting that variation in the flavonoid profile might significantly affect biological antioxidant activity.

### 3.5 Correlation analysis between chemical markers and antioxidant activity

**Figure 6:**
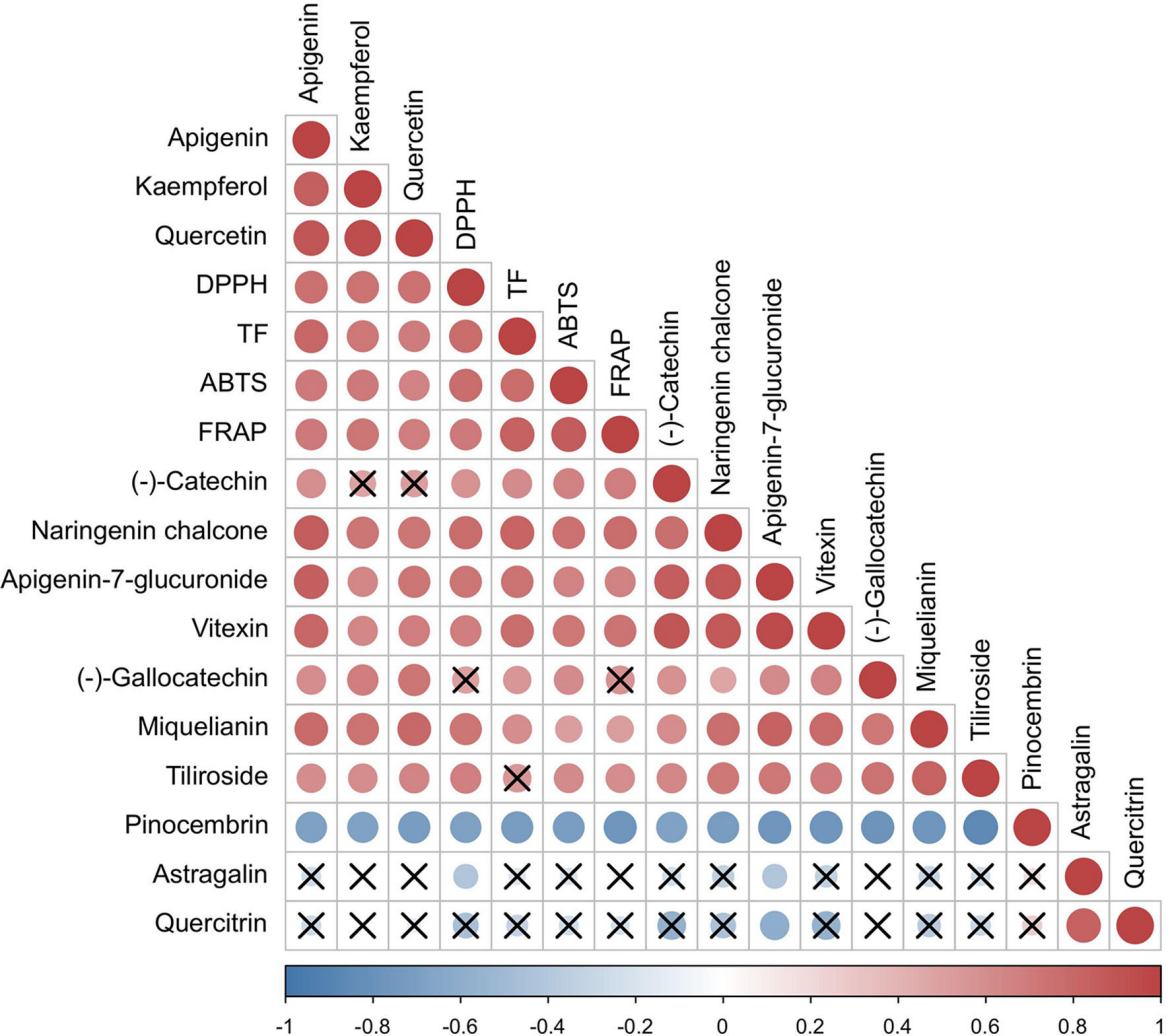
Correlation among antioxidant activities and concentration of flavonoid markers. The red colour indicates the positive correlation, the blue colour indicates the negative correlation. The “×”sign indicates that the corresponding correlation is not statistically significant.

Previous studies have shown that the antioxidant capacity of *P. petiolosa* is influenced by its flavonoid content. To gain a better understanding of the relationship between the antioxidant capacity and the variation in the concentration of individual flavonoids, we assessed the correlation between these two metrics (**Figure 6**). Remarkably, the concentration of flavonoids which were upregulated in the GZ group ((-)-catechin, naringenin chalcone, apigenin-7-glucuronide, vitexin, miquelianin, and tiliroside) were significantly positively correlated with DPPH and FRAP. Other than the above six constituents, (-)-gallocatechin level was significantly positive correlation with ABTS activity. Pinocembrin was significantly negatively correlated with the results of the DPPH, FRAP, and ABTS assays. Most of these flavonoid constituents have great potential as antioxidants. For instance, catechin, naringenin chalcone, apigenin-7-glucuronide, vitexin, miquelianin, and tiliroside have been demonstrated to exhibit efficient radical scavenging capacity and FRAP (Kashchenko et al., 2021; Rigling et al., 2021; Zhang et al., 2022). These flavonoid constituents are likely to be responsible for the higher antioxidant activities of the GZ samples in terms of DPPH, FRAP, and ABTS.

### 3.6 Correlation analysis of ecogeographic factors with chemical markers and antioxidant activity

**Figure 7:**
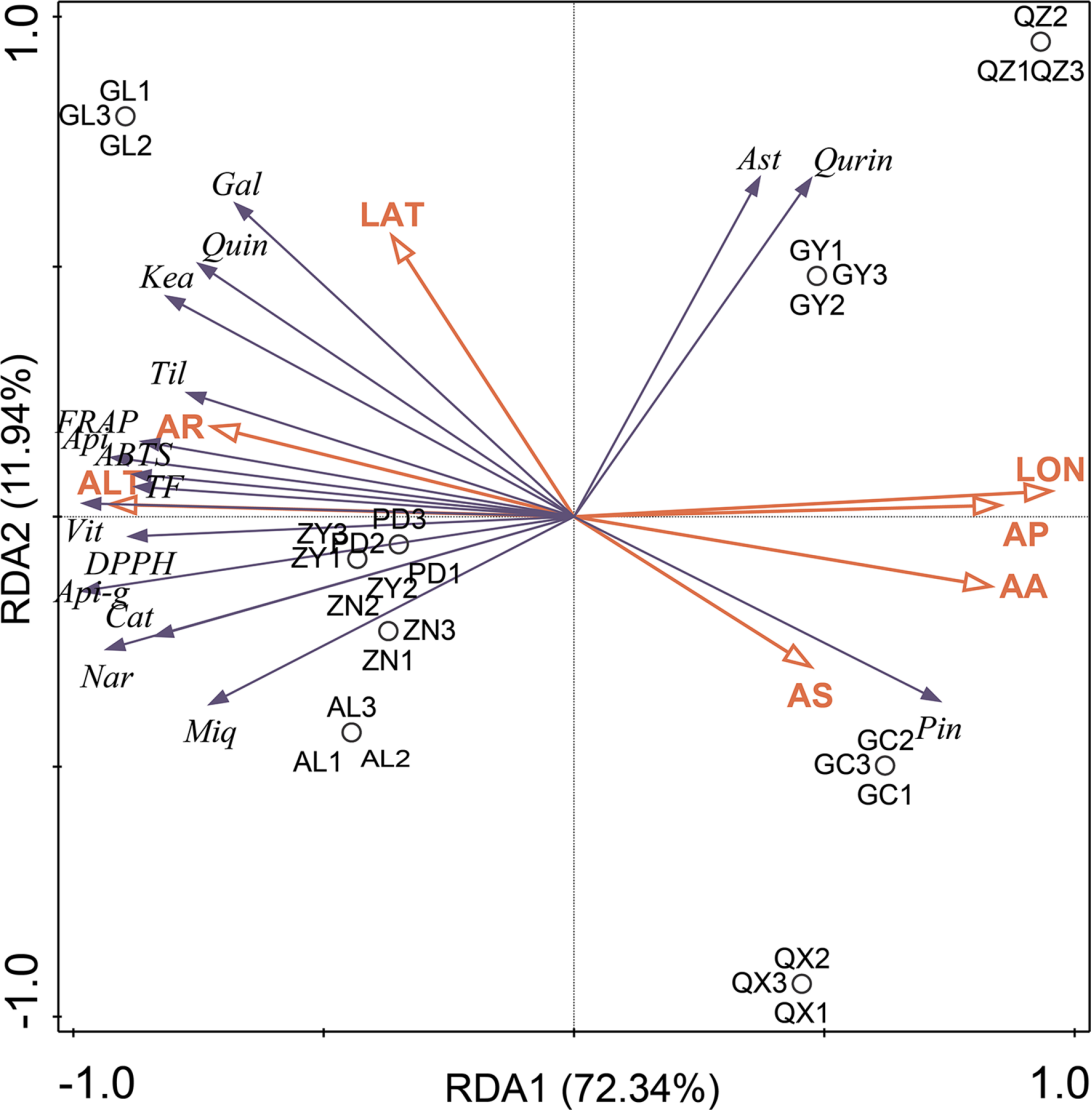
Results of RDA analysis presenting the correlation between flavonoid markers and antioxidant activities and ecogeographic parameters in each sample. Seven ecogeographic parameters were studied: (ALT), longitude (LON), latitude (LAT), average precipitation (AP), temperature (AA), sunshine (AS) and relative humidity (AR). 13 flavonoid markers were included: (-)-catechin (Cat), naringenin chalcone (Nar), apigenin-7-glucuronide (Api-g), apigenin (Api), vitexin (Vit), miquelianin (Miq), tiliroside (Til), and (-)-gallocatechin (Gal), keampferol (Kea), quercetin (Quin), astragalin (Ast), quercitrin (Qurin), pinocembrin (Pin).

Generally, the high content of functional metabolites in medicinal plants is closely related to their strong biological activity. However, the exact reason for the high metabolite content remains unclear. Some scientists have conjectured that genotypes and specific geographical factors, such as altitude, longitude, latitude, temperature, climate, and soil conditions, could greatly contribute to the biosynthesis and accumulation of these functional metabolites (Das et al., 2022; Li et al., 2022). In the present study, the relationship between the abundance of flavonoid markers and antioxidant activity of *P. petiolosa* samples from different origins and various selected ecogeographic parameters (namely altitude, longitude, latitude, average precipitation, temperature, sunshine, and relative humidity) was examined using an ordination method (redundancy analysis, RDA). In this way, the ecogeographic factors that favoured the highest flavonoid abundance and antioxidant activity in *P. petiolosa* could be determined. The results are shown in **Figure 7**. The first and second RDA axes explained 72.34% and 11.94% of the variation in the flavonoid content and antioxidant activity, respectively. Based on the length of the arrows, the most important ecogeographic parameters affecting flavonoid content and antioxidant activity of *P. petiolosa* were altitude and longitude. Generally, longitude and latitude indirectly affect medicinal plants through the influence of temperature and precipitation; therefore, their influence is important (Zhang et al., 2018). Three of the *in vitro* measures of antioxidant activity (DPPH, ABTS, and FRAP), TFC, and several flavonoid constituents (e.g. (-)-catechin, naringenin chalcone, apigenin-7-glucuronide, vitexin, miquelianin, and tiliroside), which were confirmed to be positively correlated with antioxidant activity, were positively correlated with altitude and average relative humidity through RDA analysis. This indicates that the generation and accumulation of these potential antioxidants in *P. petiolosa* depended on higher altitude and relative humidity. Additionally, significant correlations were detected between other ecogeographic parameters (e.g. average precipitation, temperature, sunshine, and longitude), flavonoid content, and antioxidant activity. Hence, a rise in several of the ecogeographical parameters studied may have a negative impact on flavonoid accumulation and antioxidant activity in *P. petiolosa*. Su et al. (2020) similarly reported that temperature, water vapour pressure, and other parameters were significantly negatively correlated with flavonoid content. The total flavonoid content in Chinese prickly ash leaves was significantly correlated with annual sunshine duration, precipitation, and relative humidity (p < 0.05). Overall, the biosynthesis and accumulation of secondary metabolites are modulated by plants to optimise their survival in response to complicated environmental conditions or abiotic stress (Ouerghemmi et al., 2016; Ribeiro et al., 2020). In our analysis, it was evident that ecogeographic factors in different geographical regions might have a significant impact on the *in vitro* antioxidant capacity and flavonoid composition.

## 4 Conclusions

In this study, a metabolomics method based on UPLC-MRM-MS was used to comprehensively quantify the 97 flavonoid components of *P. petiolosa* from different geographic origins. Multivariate statistical analysis systematically revealed that geographic origin had clear effects on the flavonoid composition and their concentrations. In view of this difference, all geographic origins could be accurately divided into two groups, allowing for the screening of potential flavonoid markers. Thirteen flavonoid markers were identified. Most of these markers were present at higher concentrations in the GZ group. In addition, the *in vitro* antioxidant activity assay suggested that the GZ group exhibited higher DPPH and ABTS free radical scavenging activity, as well as higher FRAP, than the GX group, which can be partly attributed to their distinct individual flavonoid concentrations. In summary, the GZ region was considered to be a higher-quality distribution region for *P. petiolosa*. Many ecogeographic factors, especially altitude and longitude, play important roles in the differences in antioxidant activities and flavonoid concentrations. Thus, our findings not only provide new insights into the flavonoid characterisation of *P. petiolosa* but also contribute to our understanding of how its geographical origin influences *P. petiolosa* quality as a medicinal plant. This study provides a useful basis for the further utilisation of *P. petiolosa* as natural antioxidant.

## Data availability statement

The original contributions presented in the study are included in the article/**Supplementary Material**. Further inquiries can be directed to the corresponding authors.

## Author contributions

LL and RH contributed to conception and design of the study. JC and SN established the experiments and carried out the experiment. WX, XL, YB and XZ performed the statistical analysis. JC wrote the first draft of the manuscript. LL and RH wrote sections of the manuscript. All authors contributed to the article and approved the submitted version.

## References

[B1] Al-Mekhlafi N. A. Mediani A. Ismail N. H. Abas F. Dymerski T. Lubinska-Szczygeł M.. (2021). Metabolomic and antioxidant properties of different varieties and origins of dragon fruit. Microchemical J. 160, 105687. doi: 10.1016/j.microc.2020.105687

[B2] Cheng D. Zhang Y. Gao D. Zhang H. (2014). Antibacterial and anti-inflammatory activities of extract and fractions from *Pyrrosia petiolosa* (Christ et bar.) Ching. J. Ethnopharmacol 155 (2), 1300–1305. doi: 10.1016/j.jep.2014.07.029 25077464

[B3] Cui L. Zhang Y. Shao W. Gao D. (2016). Analysis of the HPLC fingerprint and QAMS from *Pyrrosia* species. Ind. Crops Products 85, 29–37. doi: 10.1016/j.indcrop.2016.02.043

[B4] Das S. Sandeep I. S. Mohapatra P. Kar B. Sahoo R. K. Subudhi E.. (2022). A comparative study of essential oil profile, antibacterial and antioxidant activities of thirty *Piper betle* landraces towards selection of industrially important chemotypes. Ind. Crops Products 187, 115289. doi: 10.1016/j.indcrop.2022.115289

[B5] Guo X. Long P. Meng Q. Ho C. T. Zhang L. (2018). An emerging strategy for evaluating the grades of keemun black tea by combinatory liquid chromatography-orbitrap mass spectrometry-based untargeted metabolomics and inhibition effects on α-glucosidase and α-amylase. Food Chem. 246, 74–81. doi: 10.1016/j.foodchem.2017.10.14 29291881

[B6] Guo L. Wang S. Zhang J. Yang G. Zhao M. Ma W.. (2013). Effects of ecological factors on secondary metabolites and inorganic elements of *Scutellaria baicalensis* and analysis of geoherblism. Sci. China Life Sci. 56 (11), 1047–1056. doi: 10.1007/s11427-013-4562-5 24203454

[B7] Hsu C. Y. (2008). Antioxidant activity of *Pyrrosia petiolosa* . Fitoterapia 79 (1), 64–66. doi: 10.1016/j.microc.2020.105687 17855022

[B8] Huh J. Sung S. H. (2012). New flavonoid glycosides from *Pyrrosia petiolosa* and their neuroprotective activities against glutamate-induced oxidative stress in HT22 hippocampal cells. Planta Med. 78 (05), P_95. doi: 10.1055/s-0032-1307603

[B9] Kashchenko N. I. Olennikov D. N. Chirikova N. K. (2021). Metabolites of Siberian raspberries: LC-MS profile, seasonal variation, antioxidant activity and, thermal stability of rubus matsumuranus phenolome. Plants 10 (11), 2317. doi: 10.3390/plants10112317 34834680 PMC8620613

[B10] Lang T. Q. Zhang Y. Chen F. Luo G. Y. Yang W. D. (2021). Characterization of chemical components with diuretic potential from *Pyrrosia petiolosa* . J. Asian Natural Products Res. 23 (8), 764–771. doi: 10.1080/10286020.2020.1786065 32602352

[B11] Li Y. Kong D. Fu Y. Sussman M. R. Wu H. (2020a). The effect of developmental and environmental factors on secondary metabolites in medicinal plants. Plant Physiol. Biochem. 148, 80–89. doi: 10.1016/j.plaphy.2020.01.006 31951944

[B12] Li R. Sun Z. Zhao Y. Li L. Yang X. Cen J.. (2021). Application of UHPLC-Q-TOF-MS/MS metabolomics approach to investigate the taste and nutrition changes in tilapia fillets treated with different thermal processing methods. Food Chem. 356, 129737. doi: 10.1016/j.foodchem.2021.129737 33836358

[B13] Li Y. Wang X. Li C. Huang W. Gu K. Wang Y.. (2020b). Exploration of chemical markers using a metabolomics strategy and machine learning to study the different origins of *Ixeris denticulata* (Houtt.) stebb. Food Chem. 330, 127232. doi: 10.1016/j.foodchem.2020.127232 32535318

[B14] Li C. R. Yang L. X. Guo Z. F. Yang H. Zhang Y. Wang Y. M.. (2022). LC-MS-based untargeted metabolomics reveals chemical differences of cannabis leaves from different regions of China. Ind. Crops Products 176, 114411. doi: 10.1016/j.indcrop.2021.114411

[B15] Lin Y. Tang D. Liu X. Cheng J. Wang X. Guo D.. (2022). Phenolic profile and antioxidant activity of longan pulp of different cultivars from south China. LWT 165, 113698. doi: 10.1016/j.lwt.2022.113698

[B16] Liu Z. Liu B. Wen H. Tao Y. Shao Y. (2020). Phytochemical profiles, nutritional constituents and antioxidant activity of black wolfberry (*Lycium ruthenicum* murr.). Ind. Crops Products 154, 112692. doi: 10.1016/j.indcrop.2020.112692

[B17] Mahajan M. Kuiry R. Pal P. K. (2020). Understanding the consequence of environmental stress for accumulation of secondary metabolites in medicinal and aromatic plants. J. Appl. Res. Medicinal Aromatic Plants 18, 100255. doi: 10.1016/j.jarmap.2020.100255

[B18] Mais E. Alolga R. N. Wang S. L. Linus L. O. Yin X. Qi L. W. (2018). A comparative UPLC-Q/TOF-MS-based metabolomics approach for distinguishing zingiber officinale Roscoe of two geographical origins. Food Chem. 240, 239–244. doi: 10.1016/j.foodchem.2017.07.106 28946267

[B19] Moore B. D. Andrew R. L. Külheim C. Foley W. J. (2014). Explaining intraspecific diversity in plant secondary metabolites in an ecological context. New Phytol. 201 (3), 733–750. doi: 10.1111/nph.12526 24117919

[B20] Olszowy M. Dawidowicz A. L. (2016). Essential oils as antioxidants: Their evaluation by DPPH, ABTS, FRAP, CUPRAC, and β-carotene bleaching methods. Monatshefte Für Chemie-Chemical Monthly 147 (12), 2083–2091. doi: 10.1007/s00706-016-1837-0

[B21] Ouerghemmi S. Sebei H. Siracusa L. Ruberto G. Saija A. Cimino F.. (2016). Comparative study of phenolic composition and antioxidant activity of leaf extracts from three wild Rosa species grown in different Tunisia regions: *Rosa canina* l., *Rosa moschata* herrm. and *Rosa sempervirens* l. Ind. Crops Products 94, 167–177. doi: 10.1016/j.indcrop.2016.08.019

[B22] Rana A. Samtiya M. Dhewa T. Mishra V. Aluko R. E. (2022). Health benefits of polyphenols: A concise review. J. Food Biochem. 46 (10), e14264. doi: 10.1111/jfbc.14264 35694805

[B23] Ribeiro D. A. Camilo C. J. Nonato C. de F. A. Rodrigues F. F. G. Menezes I. R. A.. (2020). Influence of seasonal variation on phenolic content and *in vitro* antioxidant activity of secondatia floribunda a. DC.(Apocynaceae). Food Chem. 315, 126277. doi: 10.1016/j.foodchem.2020.126277 32004983

[B24] Rigling M. Liu Z. Hofele M. Prozmann J. Zhang C. Ni L.. (2021). Aroma and catechin profile and *in vitro* antioxidant activity of green tea infusion as affected by submerged fermentation with *Wolfiporia cocos* (Fu ling). Food Chem. 361, 130065. doi: 10.1016/j.foodchem.2021.130065 34023683

[B25] Santos M. C. B. da Silva Lima L. R. Nascimento F. R. do Nascimento T. P. Cameron L. C. Ferreira M. S. L. (2019). Metabolomic approach for characterization of phenolic compounds in different wheat genotypes during grain development. Food Res. Int. 124, 118–128. doi: 10.1016/j.foodres.2018.08.034 31466630

[B26] Shen N. Wang T. Gan Q. Liu S. Wang L. Jin B. (2022). Plant flavonoids: Classification, distribution, biosynthesis, and antioxidant activity. Food Chem. 383, 132531. doi: 10.1016/j.foodchem.2022.132531 35413752

[B27] Sheng Z. Jiang Y. Liu J. Yang B. (2021). UHPLC–MS/MS analysis on flavonoids composition in astragalus membranaceus and their antioxidant activity. Antioxidants 10 (11), 1852. doi: 10.3390/antiox10111852 34829723 PMC8614773

[B28] Su K. Zheng T. Chen H. Zhang Q. Liu S. (2020). Climate effects on flavonoid content of zanthoxylum bungeanum leaves in different development stages. Food Sci. Technol. Res. 26 (6), 805–812. doi: 10.3136/fstr.26.805

[B29] Tang C. Yang D. P. Chen J. Zhang L. X. Ding P. Xu X. J.. (2021). Geographical origin discrimination of *Amomi fructus* using an Ultra−Performance liquid Chromatography−Quadrupole Time−of−Flight mass spectrometry−based metabolomics approach combined with antioxidant activity analysis. Pharmacognosy Magazine 17 (75), 492–498. doi: 10.4103/pm.pm_59_20

[B30] Vifta R. L. Luhurningtyas F. P. (2019). Fractionation of metabolite compound from medinilla speciosa and their antioxidant activities using ABTS.+ radical cation assay. Advance Sustain. Sci. Eng. Technol. 1 (1), 0190104. doi: 10.26877/asset.v1i1.4878

[B31] Wang N. Wang J. H. Li X. Ling J. H. Li N. (2006). Flavonoids from *Pyrrosia petiolosa* (Christ) Ching: Note. J. Asian Natural Products Res. 8 (8), 753–756. doi: 10.1080/10286020500246550 17145666

[B32] Williamson G. Kay C. D. Crozier A. (2018). The bioavailability, transport, and bioactivity of dietary flavonoids: A review from a historical perspective. Compr. Rev. Food Sci. Food Saf. 17 (5), 1054–1112. doi: 10.1111/1541-4337.12351 33350159

[B33] Xiao W. Peng Y. Tan Z. Lv Q. Chan C. Yang J.. (2017). Comparative evaluation of chemical profiles of *Pyrrosiae folium* originating from three pyrrosia species by HPLC-DAD combined with multivariate statistical analysis. Molecules 22 (12), 2122. doi: 10.3390/molecules22122122 29194397 PMC6150016

[B34] Yang C. Shi J. G. Mo S. Y. Yang Y. C. (2003). Chemical constituents of *Pyrrosia petiolosa* . J. Asian Natural products Res. 5 (2), 143–150. doi: 10.1080/1028602031000066843 12765199

[B35] Zhang X. Li X. Su M. Du J. Zhou H. Li X.. (2020). A comparative UPLC-Q-TOF/MS-based metabolomics approach for distinguishing peach (*Prunus persica* (L.) batsch) fruit cultivars with varying antioxidant activity. Food Res. Int. 137, 109531. doi: 10.1016/j.foodres.2020.109531 33233161

[B36] Zhang J. Wang N. Zhang W. Chen W. Yu H. (2022). UPLC-Q-Exactive-MS based metabolomics reveals chemical variations of three types of insect teas and their *in vitro* antioxidant activities. LWT - Food Sci. Technol. 160, 113332. doi: 10.1016/j.lwt.2022.113332

[B37] Zhang X. D. Yu Y. G. Yang D. F. Qi Z. C. Liu R. Z. Deng F. T.. (2018). Chemotaxonomic variation in secondary metabolites contents and their correlation between environmental factors in *Salvia miltiorrhiza* bunge from natural habitat of China. Ind. Crops Products 113, 335–347. doi: 10.1016/j.indcrop.2018.01.043

[B38] Zhou F. Wang X. (2022). *Pyrrosia petiolosa* extract ameliorates ethylene glycol-induced urolithiasis in rats by inhibiting oxidative stress and inflammatory response. Dis. Markers 11, 1913067. doi: 10.1155/2022/1913067 PMC9374559 35968503

