# Supplementary material for: Variation in flavonoid and antioxidant activities of Pyrrosia petiolosa (Christ) Ching from different geographic origins
Source: Front Plant Sci. 2023 Apr 14;14:1173489. doi: 10.3389/fpls.2023.1173489 (PMC10140315; doi:10.3389/fpls.2023.1173489)
Supplement: Supplementary file 2 [file DataSheet_2.docx]

Supplementary Material

Variation in flavonoid and antioxidant activities of *Pyrrosia petiolosa* (Christ) Ching from different geographic origins

F **Jianhua Chen^1^** **^†^, Shan Ning ^2^** **^†^, Xuan Lu ^1^, Wei Xiang ^3^, Xiao Zhou ^1^, Yuanyuan Bu ^1^, Liangbo Li ^1*^, Rongshao Huang ^1*^**

* Correspondence: Liangbo Li (llb100@126.com) & Rongshao Huang (hrshao802@163.com)

# Supplementary Figures and Tables

## Supplementary Figures

#
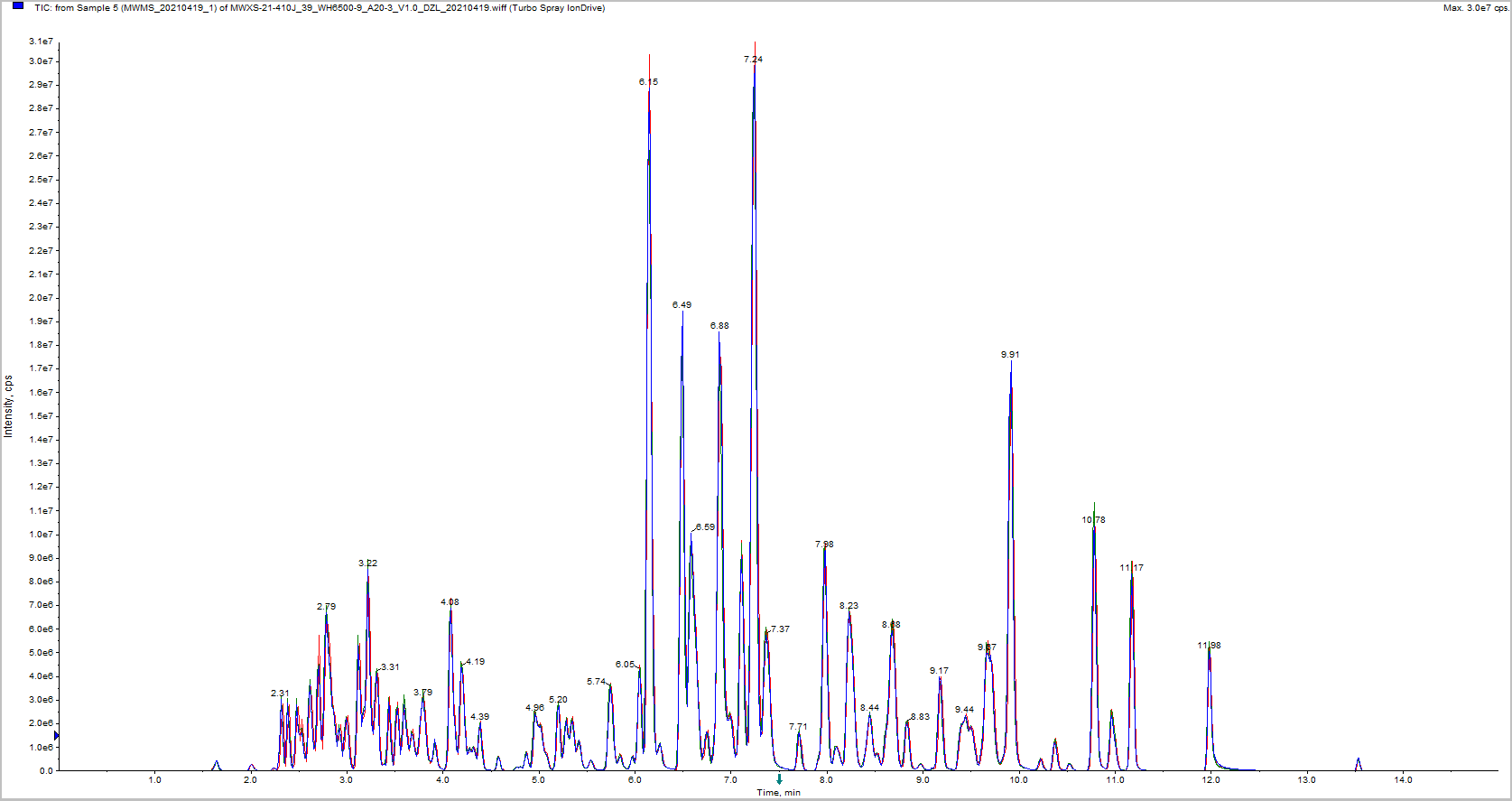


# Supplementary Figure 1. Total ion current of mixture sample.

## Supplementary Table

Supplementary Table 1. Information and ecogeographic factors of sampling site location

| Code | Place of collection^a^ | Geographical coordinates | Altitude (m) | Average temperature (℃) | Precipitation (mm) | Sunshine (hour) | Annual relative humidity (%) |
| --- | --- | --- | --- | --- | --- | --- | --- |
| ZN | Zhenning, GZ | 105.75◦N; 26.07◦E | 1251 | 16.01 | 1364 | 1161 | 79.86 |
| GL | Guanglin, GZ | 105.58◦N;  25.9 ◦E | 1142 | 16.89 | 1299 | 1136 | 79.62 |
| ZY | Ziyun, GZ | 106.06◦N; 25.85◦E | 1122 | 16.22 | 1538 | 1128 | 80.82 |
| PD | Puding, GZ | 105.71◦N; 26.38◦E | 1140 | 15.84 | 1144 | 1236 | 78.85 |
| AL | Anlong, GZ | 105.50◦N; 25.25◦E | 1117 | 16.13 | 1301 | 1672 | 83.82 |
| QZ | Quanzhou, GX | 111.05◦N; 25.98◦E | 201 | 18.97 | 2533 | 1434 | 76.23 |
| GY | Guanyang, GX | 111.11◦N；25.61◦E | 263 | 18.46 | 1906 | 1276 | 78.95 |
| GC | Gongcheng, GX | 110.95◦N; 25.18◦E | 295 | 20.56 | 1728 | 1508 | 74.68 |
| QX | Qixing, GX | 110.31◦N; 25.18◦E | 166 | 20.20 | 2535 | 1357 | 73.57 |

Note: GZ is Guizhou province, China, and GX is Guangxi province, China

Supplementary Table 2. The linear equations and correlation coefficients of the standard curves for the flavonoid components tested.

| **Compounds** | **Class** | **RT** | **Equation** | **r** | **Weighting** | **LLOQ** | **ULOQ** |
| --- | --- | --- | --- | --- | --- | --- | --- |
| (-)-Catechin | Flavanols | 2.25 | y = 1546.07908 x - 4935.61711 | 0.99878 | 1 / x | 20 | 2000 |
| (-)-Epicatechin | Flavanols | 2.47 | y = 2590.70498 x - 5384.11019 | 0.99867 | 1 / x | 10 | 2000 |
| (-)-Gallocatechin | Flavanols | 1.65 | y = 6015.69670 x - 21830.64908 | 0.9986 | 1 / x | 10 | 2000 |
| 2-Hydroxydaidzein | Isoflavanones | 3.61 | y = 26677.19936 x - 8027.97914 | 0.99675 | 1 / x | 5 | 2000 |
| 3-Methoxypuerarin | Flavone glycosides | 2.40 | y = 13780.72978 x - 3.29316e4 | 0.998 | 1 / x | 1 | 2000 |
| 5,7,3,4-Tetramethoxyflavone | Flavones | 6.15 | y = 1.05812e6 x - 9.55907e4 | 0.99349 | 1 / x | 0.5 | 2000 |
| 5-O-Demethylnobiletin | Flavones | 8.70 | y = 1.31687e5 x - 8978.99654 | 0.99715 | 1 / x | 1 | 2000 |
| 7,4-Di-O-methylapigenin | Flavones | 9.71 | y = 1.49316e5 x + 8022.30411 | 0.99721 | 1 / x | 1 | 2000 |
| Acacetin | Flavones | 7.13 | y = 6.79229e4 x - 11676.84875 | 0.99689 | 1 / x | 5 | 2000 |
| Afzelechin | Flavanols | 2.59 | y = 3218.50593 x - 2128.42772 | 0.99913 | 1 / x | 10 | 2000 |
| Afzelin | Flavonols | 3.53 | y = 3.30016e4 x + 2045.49916 | 0.99239 | 1 / x | 1 | 2000 |
| Amentoflavone | Biflavonoids | 5.74 | y = 4.84674e4 x - 11410.17474 | 0.99802 | 1 / x | 1 | 2000 |
| Apigenin | Flavones | 5.04 | y = 14202.04572 x - 3721.54018 | 0.99797 | 1 / x | 5 | 2000 |
| Apigenin 7-glucoside | Flavones | 3.23 | y = 24477.96662 x - 6.87904e4 | 0.99658 | 1 / x | 1 | 2000 |
| Apigenin-7-glucuronide | Flavones | 3.24 | y = 18435.95554 x - 1.39715e5 | 0.99762 | 1 / x | 1 | 2000 |
| Astilbin | Flavanonols | 3.01 | y = 9115.19585 x - 3.48749e4 | 0.99852 | 1 / x | 10 | 2000 |
| Astragalin | Flavonols | 3.13 | y = 26922.09397 x - 8093.35836 | 0.99159 | 1 / x | 1 | 2000 |
| Avicularin | Flavonols | 3.12 | y = 17444.54864 x - 4967.71379 | 0.99549 | 1 / x | 1 | 2000 |
| Baimaside | Flavonols | 2.49 | y = 3.27237e4 x - 1.22608e5 | 0.99785 | 1 / x | 1 | 2000 |
| Baohuoside I | Flavonols | 7.27 | y = 8.98364e4 x - 4784.28392 | 0.99647 | 1 / x | 1 | 2000 |
| Benzylideneacetophenone | Chalcones | 9.44 | y = 7.77988e4 x + 7160.30694 | 0.99543 | 1 / x | 1 | 2000 |
| Calycosin | Isoflavanones | 4.40 | y = 3.94757e4 x - 1880.23871 | 0.99617 | 1 / x | 1 | 2000 |
| Chrysin | Flavones | 7.05 | y = 6867.78551 x - 1112.24444 | 0.99779 | 1 / x | 5 | 2000 |
| Chrysosplenetin | Flavones | 7.13 | y = 29447.00777 x + 3548.26923 | 0.9945 | 1 / x | 1 | 2000 |
| Cynaroside | Flavones | 2.87 | y = 20414.39887 x - 5.80536e4 | 0.99837 | 1 / x | 5 | 2000 |
| Demethyltexasin | Isoflavanones | 3.56 | y = 4471.20622 x - 16357.89431 | 0.99571 | 1 / x | 5 | 2000 |
| Dihydrokaempferol | Flavanonols | 3.70 | y = 14811.92308 x - 4.15315e4 | 0.99887 | 1 / x | 5 | 2000 |
| Dihydromyricetin | Flavanonols | 2.65 | y = 10490.41126 x - 3.36470e4 | 0.9984 | 1 / x | 5 | 2000 |
| Diosmetin | Flavones | 5.30 | y = 6.43059e4 x - 3311.95610 | 0.99452 | 1 / x | 1 | 2000 |
| Echinatin | Chalcones | 5.00 | y = 6448.71394 x + 1459.87413 | 0.99797 | 1 / x | 5 | 2000 |
| Engeletin | Flavanonols | 3.33 | y = 6916.18487 x - 4.92824e4 | 0.99807 | 1 / x | 5 | 2000 |
| Eriocitrin | Flavanones | 2.72 | y = 3.53744e4 x - 1.16527e5 | 0.99608 | 1 / x | 1 | 2000 |
| Eriodictyol | Flavanones | 4.24 | y = 7224.69055 x - 23256.94074 | 0.99892 | 1 / x | 5 | 2000 |
| Eupatorin | Flavones | 6.65 | y = 6.98377e4 x - 9154.16636 | 0.99692 | 1 / x | 1 | 2000 |
| Fisetin | Flavonols | 3.59 | y = 9182.58962 x - 5118.11979 | 0.99519 | 1 / x | 5 | 2000 |
| Formononetin | Isoflavanones | 6.06 | y = 1.23434e5 x - 8867.37517 | 0.99455 | 1 / x | 1 | 2000 |
| Galangin | Flavones | 7.30 | y = 1869.37796 x - 777.19229 | 0.99653 | 1 / x | 10 | 2000 |
| Genkwanin | Flavones | 7.24 | y = 5.10579e4 x + 6239.57035 | 0.99247 | 1 / x | 5 | 2000 |
| Hesperetin | Flavanones | 5.35 | y = 3.01333e4 x - 3937.55688 | 0.99631 | 1 / x | 0.5 | 2000 |
| Homoplantaginin | Flavones | 3.31 | y = 4.52539e4 x - 9330.07404 | 0.99344 | 1 / x | 1 | 2000 |
| Hydroxysafflor yellow A | - | 2.04 | y = 1747.03819 x - 7423.75992 | 0.99829 | 1 / x | 5 | 2000 |
| Hyperoside | Flavonols | 2.83 | y = 95.16410 x - 1485.21317 | 0.99848 | 1 / x | 1 | 2000 |
| Isoliquiritigenin | Chalcones | 5.77 | y = 5.10410e4 x - 2384.56594 | 0.99508 | 1 / x | 1 | 2000 |
| Isomangiferin | Xanthones | 2.40 | y = 22338.76563 x - 5.69309e4 | 0.99828 | 1 / x | 1 | 2000 |
| Isoorientin | Flavone glycosides | 2.55 | y = 18787.14959 x - 18864.72766 | 0.99425 | 1 / x | 5 | 2000 |
| Isorhamnetin | Flavonols | 5.37 | y = 3.96295e4 x - 3.69561e4 | 0.9982 | 1 / x | 1 | 2000 |
| Isorhamnetin 3-O-glucoside | Flavonols | 3.19 | y = 3.16036e4 x - 1.29506e5 | 0.99572 | 1 / x | 1 | 2000 |
| Kaempferitrin | Flavonols | 2.79 | y = 10364.33032 x - 665.80241 | 0.9926 | 1 / x | 1 | 2000 |
| Kaempferol | Flavonols | 5.19 | y = 1793.42402 x - 5344.16206 | 0.99904 | 1 / x | 20 | 2000 |
| Kaempferol 3-neohesperidoside | Flavonols | 2.78 | y = 14785.27137 x - 11692.89093 | 0.99502 | 1 / x | 5 | 2000 |
| Kurarinone | Other flavonoids | 7.38 | y = 4.67291e4 x - 14789.59569 | 0.9986 | 1 / x | 5 | 2000 |
| Laricitrin | Flavonols | 4.35 | y = 21180.05332 x - 4.87950e4 | 0.99692 | 1 / x | 5 | 2000 |
| Licoisoflavone A | Isoflavanones | 8.10 | y = 27795.44701 x - 861.82066 | 0.99766 | 1 / x | 1 | 2000 |
| Isoliquiritigenin | Chalcones | 5.77 | y = 5.10410e4 x - 2384.56594 | 0.99508 | 1 / x | 1 | 2000 |
| Luteolin | Flavones | 4.29 | y = 23859.80739 x - 9.59197e4 | 0.9989 | 1 / x | 5 | 2000 |
| Mangiferin | Xanthones | 2.32 | y = 12214.68472 x - 4.22020e4 | 0.99832 | 1 / x | 5 | 2000 |
| Miquelianin | Flavonols | 2.84 | y = 25088.78077 x - 2.00612e5 | 0.99527 | 1 / x | 5 | 2000 |
| Morusin | Other flavonoids | 11.01 | y = 3.35233e4 x - 316.79004 | 0.99599 | 1 / x | 5 | 2000 |
| Myricetin | Flavonols | 3.55 | y = 16835.62596 x - 7.75799e4 | 0.99807 | 1 / x | 5 | 2000 |
| Narcissin | Flavones | 2.99 | y = 24378.38001 x - 1.95664e5 | 0.99783 | 1 / x | 5 | 2000 |
| Naringenin chalcone | Chalcones | 5.03 | y = 17725.38139 x - 20337.69144 | 0.99833 | 1 / x | 5 | 2000 |
| Naringenin-7-glucoside | Flavanones | 3.28 | y = 18525.20726 x - 4.49532e4 | 0.99841 | 1 / x | 5 | 2000 |
| Naringin Dihydrochalcone | Chalcones | 3.53 | y = 3.13378e4 x - 2.51382e5 | 0.99739 | 1 / x | 1 | 2000 |
| Nicotiflorin | Flavones | 2.94 | y = 16254.67033 x - 6932.16960 | 0.99534 | 1 / x | 1 | 2000 |
| Nobiletin | Flavones | 7.22 | y = 3.42782e5 x + 9294.08554 | 0.99264 | 1 / x | 1 | 2000 |
| Ononin | Isoflavanones | 3.74 | y = 9903.93578 x - 2350.27891 | 0.99787 | 1 / x | 1 | 2000 |
| Orientin | Flavone glycosides | 2.61 | y = 20624.06405 x - 7448.58262 | 0.996 | 1 / x | 1 | 2000 |
| Oroxin A | Flavones | 3.68 | y = 24422.16082 x - 7721.19355 | 0.99767 | 1 / x | 1 | 2000 |
| Phloretin | Chalcones | 4.97 | y = 6.49980e4 x - 5.52231e4 | 0.99735 | 1 / x | 1 | 2000 |
| Pinocembrin | Flavanones | 7.22 | y = 5306.16403 x - 127.11712 | 0.99661 | 1 / x | 1 | 2000 |
| Poncirin | Flavanones | 4.20 | y = 4.99765e4 x - 8429.81523 | 0.99594 | 1 / x | 1 | 2000 |
| Procyanidin B2 | Anthocyanins | 2.30 | y = 2668.47708 x - 12026.42596 | 0.99851 | 1 / x | 5 | 2000 |
| Prunetin | Isoflavanones | 7.41 | y = 16075.00821 x - 4424.36623 | 0.99742 | 1 / x | 10 | 2000 |
| Puerarin | Isoflavanones | 2.33 | y = 3.54223e4 x - 1.12448e5 | 0.99802 | 1 / x | 1 | 2000 |
| Quercetin | Flavonols | 4.33 | y = 26830.84334 x - 9.78586e4 | 0.99914 | 1 / x | 5 | 2000 |
| Quercetin 3-O-(6-galloyl)-β-D-galactopyranoside | Flavonols | 2.70 | y = 18535.86796 x - 3.93753e4 | 0.99901 | 1 / x | 1 | 2000 |
| Quercimeritrin | Flavonols | 2.82 | y = 1094.09681 x + 176.72554 | 0.99214 | 1 / x | 1 | 2000 |
| Quercitrin | Flavonols | 3.19 | y = 18986.09690 x - 1.56716e5 | 0.99737 | 1 / x | 1 | 2000 |
| Robinin | Flavonols | 2.50 | y = 24064.74585 x - 10997.37823 | 0.99502 | 1 / x | 1 | 2000 |
| Rutin | Flavonols | 2.72 | y = 4.38145e4 x - 3.66148e5 | 0.9945 | 1 / x | 1 | 2000 |
| Sakuranetin | Flavones | 7.10 | y = 4.54580e4 x - 2813.78098 | 0.99794 | 1 / x | 1 | 2000 |
| Schaftoside | Flavone glycosides | 2.48 | y = 3525.73288 x - 8676.52010 | 0.99333 | 1 / x | 5 | 2000 |
| Scutellarein | Flavones | 3.81 | y = 5613.46633 x - 2225.35563 | 0.99238 | 1 / x | 5 | 2000 |
| Scutellarein tetramethyl ether | Flavones | 7.25 | y = 8.28515e5 x - 9.79107e4 | 0.99884 | 1 / x | 5 | 2000 |
| Scutellarin | Flavones | 2.85 | y = 12407.36152 x - 1658.68893 | 0.99256 | 1 / x | 1 | 2000 |
| Sieboldin | Chalcones | 3.18 | y = 4.64170e4 x - 4.34589e4 | 0.99378 | 1 / x | 20 | 2000 |
| Silychristin | Flavanonols | 3.78 | y = 8594.95483 x - 3180.63515 | 0.99419 | 1 / x | 1 | 2000 |
| Sinensetin | Flavones | 6.49 | y = 7.12176e5 x - 9.12810e4 | 0.9916 | 1 / x | 0.5 | 2000 |
| Spinosin | Flavone glycosides | 2.68 | y = 3273.52532 x - 20932.10404 | 0.99862 | 1 / x | 10 | 2000 |
| Tangeretin | Flavones | 7.98 | y = 3.97632e5 x - 5405.20405 | 0.99111 | 1 / x | 1 | 2000 |
| Taxifolin | Flavanonols | 3.18 | y = 7483.24649 x - 6.08118e4 | 0.99746 | 1 / x | 5 | 2000 |
| Taxifolin 7-O-rhamnoside | Flavanonols | 2.66 | y = 3141.12524 x - 3.10293e4 | 0.99397 | 1 / x | 10 | 2000 |
| Tectochrysin | Flavones | 9.66 | y = 1.68169e5 x + 13336.63999 | 0.9961 | 1 / x | 1 | 2000 |
| Tectorigenin | Isoflavanones | 5.21 | y = 5428.29619 x - 938.99140 | 0.99792 | 1 / x | 5 | 2000 |
| Tiliroside | Flavonols | 4.09 | y = 5.99855e4 x + 11110.76769 | 0.99456 | 1 / x | 0.5 | 2000 |
| Tricin | Flavones | 4.09 | y = 3.48949e4 x - 7270.22294 | 0.99467 | 1 / x | 1 | 2000 |
| Vitexin | Flavone glycosides | 2.79 | y = 5.27513e4 x - 1.96615e5 | 0.99226 | 1 / x | 1 | 2000 |

Note: RT: Retention time; r: Coefficient of Correlation; LLOQ (nmol/L)：Lower limit of quantification；ULOQ (nmol/L)：Upper limit of quantification.

**Supplementary Table 3.** Differentiating metabolites between GZ versus GX group.

| No. | Compounds | VIP | Fold change | p-value |
| --- | --- | --- | --- | --- |
| 1 | Vitexin | 1.772361 | 23.199 | 1.93E-16 |
| 2 | Apigenin-7-glucuronide | 1.718754 | 15.717 | 2.36E-13 |
| 3 | Apigenin | 1.64521 | 9.6971 | 6.13E-10 |
| 4 | Naringenin chalcone | 1.631203 | 4.0676 | 5.28E-10 |
| 5 | Kaempferol | 1.477465 | 2.4528 | 4.52E-07 |
| 6 | Pinocembrin | 1.436387 | 0.14181 | 5.14E-06 |
| 7 | Tiliroside | 1.426558 | 2.9221 | 1.13E-05 |
| 8 | (-)-Catechin | 1.37345 | 2.0136 | 1.24E-05 |
| 9 | Quercetin | 1.286596 | 2.223 | 9.54E-05 |
| 10 | (-)-Gallocatechin | 1.26563 | 3.0548 | 8.05E-05 |
| 11 | Astragalin | 1.118677 | 0.4567 | 0.00083267 |
| 12 | Quercitrin | 1.052084 | 0.31887 | 0.0017146 |
| 13 | Miquelianin | 1.029541 | 2.1545 | 0.001568 |
